# Up- or Downregulation of Melanin Synthesis Using Amino Acids, Peptides, and Their Analogs

**DOI:** 10.3390/biomedicines8090322

**Published:** 2020-09-01

**Authors:** Yong Chool Boo

**Affiliations:** 1 Department of Molecular Medicine, School of Medicine, Kyungpook National University, 680 Gukchaebosang-ro, Jung-gu, Daegu 41944, Korea; ycboo@knu.ac.kr; Tel.: +82-53-420-4946; 2 BK21 Plus KNU Biomedical Convergence Program, Kyungpook National University, Daegu 41944, Korea; 3 Cell and Matrix Research Institute, Kyungpook National University, Daegu 41944, Korea

**Keywords:** pigmentation, melanin, peptide, amino acid, tyrosinase, inhibitor, melanocortin 1 receptor, agonist, antagonist, melanogenesis, melanosome biogenesis, autophagy

## Abstract

Harmonious synthesis and distribution of melanin in the skin contribute to the expression of beauty and the maintenance of health. When skin pigmentary disorders occur because of internal or external factors or, when there is a need to artificially increase or reduce the pigmentation level of the skin for aesthetic or therapeutic purposes, various pharmacological therapies are applied but the results are not always satisfactory. Studies have been conducted to improve the efficacy and safety of these treatment strategies. In this review, we present the latest studies regarding peptides and related compounds that may be useful in artificially increasing or reducing skin melanin levels. Certain analogs of α-melanocyte stimulating hormone (MSH) and oligopeptides with the sequences derived from the hormone were shown to promote melanin synthesis in cells and in vivo models. Various amino acids, peptides, their analogs, and their hybrid compounds with other chemical moieties were shown to inhibit tyrosinase (TYR) catalytic activity or downregulate TYR gene expression. Certain peptides were shown to inhibit melanosome biogenesis or induce autophagy, leading to decreased pigmentation. In vivo and clinical evidence are available for some compounds, including [Nle^4^-_D_-Phe^7^]-α-MSH, glutathione disulfide, and glycinamide hydrochloride. For many other compounds, additional studies are required to verify their efficacy and safety in vivo and in clinical trials. The accumulating information regarding pro- and antimelanogenic activity of peptides and related compounds will lead to the development of novel drugs for the treatment of skin pigmentary disorders.

## 1. Introduction

Melanin plays an important role in the appearance of skin color, protection against ultraviolet (UV) radiation, and maintenance of homeostasis in many organs [1,2]. Both over- and underproduction of melanin are a major research theme in cosmetology and dermatology, not only from the aesthetic viewpoint pursuing a harmonious skin tone, but also from a medical viewpoint preventing and treating various skin diseases [3,4,5,6,7]. 

Melanin synthesis begins with tyrosinase (TYR)-catalyzed oxidation of _L_-Tyr or _L_-dihydroxyphenylalanine (DOPA), followed by the production of pheomelanin or eumelanin depending on whether conjugation reactions with _L_-Cys or glutathione (γ-Glu-Cys-Gly) are included in the intermediate process [8,9,10]. Proopiomelanocortin (POMC)-derived peptide hormones, such as α-melanocyte stimulating hormone (MSH), β-MSH, and adrenocorticotrophic hormone (ACTH), induce the expression of many key enzymes involved in melanin synthesis, including TYR, tyrosinase-related protein 1 (TYRP1), and dopachrome tautomerase (DCT) [3,11]. 

As numerous amino acids and peptides directly and indirectly participate in the melanin synthesis process, it is reasonably assumed that the process could be artificially regulated by certain structurally related compounds. In this review, we discuss recent studies on natural or synthetic peptides and related compounds that have been reported to increase or decrease melanin synthesis in vitro and in vivo. Some of these compounds may be useful in artificially up- or downregulating melanogenesis for the purpose of aesthetics or therapeutics. Hopefully, this review will assist researchers in their goal of discovering substances that regulate melanin synthesis and the industrial or medical application of such substances. 

In this review, three-letter or one-letter codes for amino acids are used: alanine, Ala, A; arginine, Arg, R; asparagine, Asn, N; aspartic acid, Asp, D; cysteine, Cys, C; glutamic acid, Glu, E; glutamine, Gln, Q; glycine, Gly, G; histidine, His, H; isoleucine, Ile, I; leucine, Leu, L; lysine, Lys, K; methionine, Met, M; phenylalanine, Phe, F; proline, Pro, P; serine, Ser, S; threonine, Thr, T; tryptophan, Trp, W; tyrosine, Tyr, Y; valine, Val, V; norleucine, Nle. _L_- and _D_- are used to indicate stereoisomers of each amino acid (except glycine). 

## 2. Melanin and Pigmentation 

Melanin is a polymeric brown or dark pigment produced by melanocytes and distributed throughout the skin, hair, eye, and other tissues [10,12]. It plays an important function in maintaining epidermal homeostasis [1,2]. Melanin absorbs UV radiation and dissipates most of the absorbed energy as heat, thus protecting the skin [13]. The photoprotective effects of melanin are evidenced by the lower incidence of malignant melanoma in dark-skinned compared with light-skinned people [4]. In an in vitro study, we demonstrated that small interfering RNAs targeting TYR decreased melanin content and melanocyte viability following UV light exposure [14]. Skin’s UV protection capability may be aided by external use of natural products that can act as UV absorbers, promoters of melanin synthesis, antioxidants, and anti-inflammatory agents [15].

The number of melanocytes per unit area of skin is not significantly different between individuals, even if they exhibit different skin color. However, melanocytes derived from different skin color groups show different melanogenic activity [12,16] and there is a close relationship between melanogenic activity and human skin color [17,18]. The vertical and horizontal distribution of melanin, as well as aggregation and dispersion of melanin in the skin affects skin color [19,20]. Skin color is largely associated with genetic mutations in the solute carrier proteins genes, SLC24A5 and SLC45A2 [21,22]. Single nucleotide polymorphisms in these genes and the resulting changes in activity of the encoded potassium-dependent, sodium-calcium exchangers affect the biogenesis of melanosomes as well as melanogenesis in melanocytes [23,24]. 

Disrupted melanin metabolism can cause skin pigmentary disorders, which can be congenital or acquired, temporary or permanent, restricted to skin or systemic, and hypo- or hyperpigmented [3,5]. Hyperpigmentation occurs when melanin synthesis is abnormally increased in response to certain stimulating factors [25]. It can occur as a result of inflammatory reactions caused by pathophysiological and physicochemical factors, or as a change accompanying intrinsic or photo-aging of the skin [26]. Hypopigmentation occurs when melanin synthesis is abnormally decreased by genetic or epigenetic variations, as in the cases of albinism and vitiligo [27,28]. Even though skin pigmentary disorders are not life-threatening, they can cause mental stress and diminish life quality [29]. Therefore, there is a great need to develop an effective treatment for unwanted hypo- and hyperpigmentation [30,31,32]. 

## 3. Melanogenesis and Key Regulators

Melanin is synthesized and stored in melanosomes, lysosome-related organelles in epidermal melanocytes, which lie at the junction of the dermis and epidermis [10,33]. A number of enzymes, such as TYR, TYRP1, and DCT, are involved in melanin synthesis [34,35]. TYR catalyzes the initial step of melanin synthesis, which is the oxidation of _L_-Tyr or _L_-DOPA to DOPAquinone [36]. The subsequent reactions vary depending on the presence or absence of thiol compounds, such as _L_-Cys and glutathione, and results in the production of reddish-yellow pheomelanin or brownish black eumelanin [9]. The addition of _L_-Cys or glutathione to DOPAquinone is followed by the subsequent transformation and polymerization to the final product, pheomelanin. In the absence of thiol compounds, DOPAquinone is oxidized to form DOPAchrome, which is then converted to 5,6-dihydroxyindole (DHI) or 5,6-dihydroxyindole-2-carboxylic acid (DHICA). Polymerization of DHI and DHICA and their quinones leads to eumelanin production. Melanin synthesis in the skin is affected by diverse factors including genetic background, hormonal changes, nutritional status, and environmental conditions [3,37]. 

The melanocortins are a group of peptide hormones derived from the posttranslational cleavage of the POMC gene product catalyzed by prohormone convertases, which include ACTH, α-MSH, β-MSH, and three γ-MSH isotypes [38]. The melanocortins show different binding affinities for each of the five melanocortin receptors (MC1R–MC5R), which are expressed in a tissue-specific fashion [39]. This central melanocortin system is a main subject not only in dermatology, but in other disciplines as it is involved in various pathways including pigmentation, lipolysis, food intake, thermogenesis, sexual behavior, memory, and inflammatory response [40,41].

ACTH, α-MSH, and β-MSH are agonists of the melanocortin 1 receptor (MC1R), a G protein coupled receptor [3,11]. α-MSH is a 13 amino acid peptide with the sequence, Ac-Ser-Tyr-Ser-Met-Glu-His-Phe-Arg-Trp-Gly-Lys-Pro-Val-NH_2_ [8,10]. Binding of agonists, such as α-MSH, to MC1R at the plasma membrane of melanocytes leads to the activation of adenylate cyclase (AC) resulting in the production of cyclic adenosine monophosphate (cAMP). Subsequently, protein kinase A (PKA) is activated and in turn phosphorylates cAMP response element-binding protein (CREB). In the nucleus, phospho-CREB binds to cAMP response element (CRE) on the promoter of microphthalmia-associated transcription factor (MITF) in DNA and induces the mRNA expression of MITF [42,43]. 

MITF plays a primary role in inducing melanogenic enzyme gene expression in response to various stimuli [3]. In addition to the α-MSH/MC1R/cAMP/PKA/CREB pathway described above, the stem cell factor (SCF)/receptor tyrosine kinase protein c-Kit /mitogen-activated protein kinases (MAPK) pathway, and WNT/frizzled/glycogen synthase kinase (GSK) 3β/β-catenin pathway can also activate MITF [44,45]. Other intracellular signaling pathways, such as phospholipase C (PLC)/diacylglycerol (DAG)/protein kinase C (PKC) β cascade, and nitric oxide (NO)/cGMP/protein kinase G (PKG) cascade are also involved in the regulation of melanogenesis [46,47]. 

The agouti signaling protein (ASP) is an antagonist of MC1R that inhibits the binding of agonists, such as α-MSH, in a competitive manner, and thereby suppresses melanogenesis [48]. Melanin-concentrating hormone (MCH) is a cyclic 19-amino acid hypothalamic peptide involved in the regulation of feeding behavior, sleep-wake cycle, and energy balance [49]. In melanocytes, MCH exhibits an antagonistic relationship with α-MSH and decreases melanin production [50]. Melanocyte-inhibiting factor (melanostatin, Pro-Leu-Gly-NH_2_) is a hypothalamic peptide hormone derived from the hormone oxytocin that elicits multiple effects including the inhibition of α-MSH release [51]. Melatonin, a hormone synthesized and released from the pineal gland, can either attenuate or stimulate melanin synthesis depending on the situation [52,53]. 

**Figure 1:**
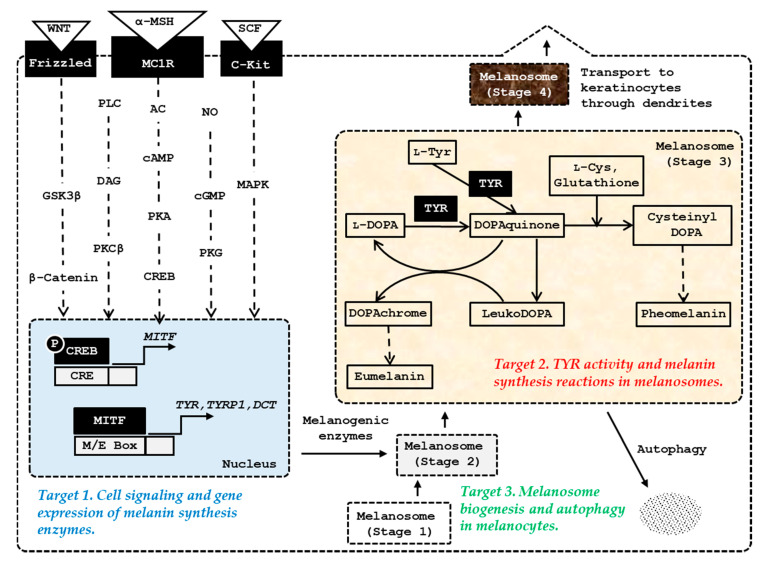
The major targets of amino acids, peptides, and their analogs for the control of skin pigmentation. Microphthalmia-associated transcription factor (MITF) plays a primary role in inducing gene expression of melanogenic enzymes, such as tyrosinase (TYR), tyrosinase-related protein 1 (TYRP1), and dopachrome tautomerase (DCT) in response to various internal and external stimuli. In addition to the α-melanocyte stimulating hormone (MSH)/ melanocortin 1 receptor (MC1R) /adenyl cyclase (AC)/ cyclic adenosine monophosphate (cAMP)/protein kinase A (PKA)/cAMP-responsive element-binding protein (CREB) pathway, the stem cell factor (SCF)/receptor tyrosine kinase protein, c-Kit/mitogen-activated protein kinases (MAPK) pathway, and WNT/frizzled/glycogen synthase kinase (GSK) 3β/β-catenin pathway can activate MITF. Other signaling pathways, such as phospholipase C (PLC)/diacylglycerol (DAG)/protein kinase C (PKC) β cascade, and nitric oxide (NO)/cGMP/protein kinase G (PKG) cascade are also involved in the activation of MITF. Melanosome biogenesis occurs through morphologically distinct stages 1, −2, −3, and −4. Melanogenic enzymes matured through post-translational modifications in endoplasmic reticulum and metal-loading in Golgi apparatus are sorted and transported to stage 2 melanosomes. Melanin is synthesized thereafter and the mature stage 4 melanosomes with accumulated melanin are transferred through dendrites to keratinocytes.

Melanosome biogenesis occurs through four morphologically distinct stages [54]. Stage 1 melanosomes appear as vacuolar multivesicular endosomes, and stage 2 melanosomes exist as ellipsoidal shapes with a striated appearance due to premelanosome protein (PMEL) fibrils. Melanogenic enzymes that are matured through post-translational modifications in endoplasmic reticulum and metal-loading in Golgi apparatus are sorted and transported to stage 2 melanosomes [55]. Thereafter, melanin is synthesized and deposited onto the PMEL fibrils inside melanosomes, resulting in stage 3 melanosomes. In stage 4 melanosomes, PMEL fibrils are fully masked by melanin and the lumen is filled with melanin. The mature stage 4 melanosomes are transferred from a single melanocyte through dendrites to the cytoplasm of 30–40 neighboring keratinocytes, resulting in the spread of melanin pigments throughout the epidermis [56]. Keratinocytes release cytokines, including α-MSH and endothelin-1, that stimulate melanocytes to promote melanogenesis and melanosome biogenesis [57]. Potential targets for the control of skin pigmentation are schematically represented in Figure 1. 

For more details regarding melanogenesis and pigmentation, please refer to the latest review articles on cell signaling pathways [47], melanosome biogenesis [45], autocrine and paracrine regulation [57], pharmacological modulation of melanogenesis [31,32], and human application [58,59]. 

## 4. Artificial Upregulation of Melanin Synthesis

In this chapter, we discuss promotion of melanin synthesis by α-MSH analogs and oligopeptides derived from the hormone sequence (Section 4.1), _L_-Tyr and _L_-DOPA (Section 4.2), and other peptide hormones (Section 4.3). Selected studies are listed in Table 1.

### 4.1. MC1R Agonist Peptides

POMC peptides such as ACTH and α-MSH exhibit mitogenic and melanogenic activity in human melanocytes [67,68]. Previous studies have used a strategy for melanoma prevention using α-MSH analogs that function as MC1R agonists to enhance eumelanin synthesis [69]. 

Sawyer et al. synthesized [Nle^4^-_D_-Phe^7^]-α-MSH, which is a linear 13-amino acid peptide with the sequence, Ac-Ser-Tyr-Ser-Nle-Glu-His-_D_-Phe-Arg-Trp-Gly-Lys-Pro-Val-NH_2_. It contains Nle (norleucine) in place of Met at the fourth position of α-MSH and _D_-Phe in place of _L_-Phe at the seventh position [60]. Compared to α-MSH or [Nle^4^]-α-MSH, [Nle^4^-_D_-Phe^7^]-α-MSH was more resistant to enzymatic degradation by serum enzymes, and it exhibited significantly increased biological activity, as determined by activation of AC and stimulation of TYR activity in mouse melanoma cells [60]. [Nle^4^-_D_-Phe^7^]-α-MSH stimulated TYR activity and inhibited the proliferation of human melanoma cells with some variation between cell lines [70]. Subcutaneous injection of [Nle^4^-_D_-Phe^7^]-α-MSH alone or in combination with UV irradiation induced tanning of human skin [71]. In a phase II trial, [Nle^4^-_D_-Phe^7^]-α-MSH increased melanin density and patient tolerance following exposure to artificial light [72].

Castrucci et al. proposed that His^6^-Phe^7^-Arg^8^-Trp^9^, or Arg^8^-Trp^9^ is the minimal message sequence of α-MSH for its melanotropic activity observed in frog and lizard skin bioassays [73,74]. Ac-α-MSH (7-10)-NH_2_ (i.e., Ac-Phe-Arg-Trp-Gly-NH_2_) was identified to be a weak α-MSH antagonist in lizards [74], but it was inactive in the other vertebrate species tested [61,74]. They further reported that Ac-Phe-Arg-Trp-Gly-NH_2_ peptide did not show an agonistic or antagonistic activity in the murine S-91 melanoma cells, but rather potentiated the α-MSH-induced increase of TYR activity [61]. Thus, the binding of Ac-Phe-Arg-Trp-Gly-NH_2_ peptide to the receptor or its bioactivity is considered highly variable among the species.

Abdel-Malek et al. identified melanin synthesis stimulating tetrapeptide analogs of α-MSH including Ac-His-_D_-Phe-Arg-Trp-NH_2_, n-Pentadecanoyl-His-_D_-Phe-Arg-Trp-NH_2_, and 4-Phenylbutyryl-His-_D_-Phe-Arg-Trp-NH_2_ [62]. These peptides stimulated melanin synthesis and increased the viability of human melanocytes under UV-irradiated conditions [62]. Jackson et al. identified pentapeptide analogs of α-MSH that function as MC1R agonists [63]. In an ex-vivo human skin tissue culture model, Bz-Gly-His-_D_-Phe-_D_-Arg-_D_-Trp-N(CH_2_CH_2_CH_3_)_2_ induced expression of MITF, TYR, and TYRP1 protein, and enhanced the activation of nuclear factor erythroid 2-related factor 2 (NRF2) following UVA-irradiation [63]. The in vivo efficacy of these melanogenic peptides has not yet been reported. 

### 4.2. _L_-Tyr and _L_-DOPA

_L_-Tyr and _L_-DOPA are substrates and allosteric modulators for TYR and their biological availability can have an impact on melanin synthesis in mammals [75,76,77]. In addition, _L_-Tyr and _L_-DOPA are known to play a hormone-like stimulatory role in stimulating melanin synthesis [64,78]. Their action mechanism or specific receptors is not clearly established [79], but they were shown to enhance the agonistic activity of melanocortins on their receptors [80], and to promote TYR protein expression via a posttranscriptional mechanism [81]. In our previous study, _L_-Tyr was shown to increase both the mRNA and protein levels of TYR, TYRP1, DCT, and MITF, and to promote melanin synthesis in human epidermal melanocytes [82].

### 4.3. Other Peptide Hormones

Vasoactive intestinal peptide (VIP) is composed of 28 amino acids and is a ligand for the G protein-coupled receptors, VIP receptor 1 and 2 [83]. Yuan et al. reported that VIP increased melanin production by increasing TYR activity and gene expression through a PKA/CREB/MITF pathway [65].

Angiotensin II, a peptide hormone that plays a role in regulating blood pressure, was shown to increase TYR activity and melanin content in human melanocytes [66]. The hormone upregulated the expression of angiotensin II receptor type 1 (AT1) and TYR, and these effects were eliminated by losartan, an AT1 antagonist, indicating that angiotensin II can play a regulatory role in melanogenesis through an AT1-dependent mechanism.

## 5. Artificial Downregulation of Melanin Synthesis

In this chapter, we discuss basic amino acids and peptides (Section 5.1), peptides isolated from plants or derived from natural protein sequences (Section 5.2), and hybrid peptides with other chemical moieties (Section 5.3) that inhibit TYR catalytic activity in vitro. We additionally discuss certain peptides that downregulate TYR gene expression or its protein level in melanocytes (Section 5.4). Finally, we discuss the peptides that inhibit melanosome biogenesis or induce autophagy in melanocytes (Section 5.5). IC_50_ is defined as the 50% inhibitory concentration. 

### 5.1. TYR Inhibitory Amino Acids, Peptides, and Their Analogs

Various amino acids and peptides are known to inhibit TYR activity and/or cellular melanin synthesis, and some of them show depigmenting effects in human skin (Table 2). 

TYR-catalyzed DOPAchrome formation and following melanin formation was inhibited by thiol compounds such as _L_-Cys and glutathione [96]. This effect may be due to the formation of a conjugate between DOPAquinone and the thiol compounds that cannot be further oxidized to form eumelanin [96,97]. 

Kahn et al. compared the effects of various _L_-amino acids on the *ortho*-dihydroxyphenolase activity of mushroom TYR [84]. Most amino acids, including _L_-Ala and _L_-Pro (330 mM), _L_-Ser and _L_-Ile (165 mM), _L_-Leu, _L_-Asn and _L_-Val (60 mM), _DL_-Asp, and _L_-Glu and _L_-Trp (15 mM), had no effect on *ortho*-dihydroxyphenolase activity as determined using _DL_-DOPA as a substrate. However, _L_-Lys, _L_-Gly, _L_-His, and _L_-Phe exhibited 50% inhibition of TYR activity at approximately 50, 65, 120, and 200 mM, respectively. The highest inhibitory effect was observed with _L_-Cys which extended an initial delay (lag period) in DOPAchrome formation and suppressed it completely at 0.3 mM. Liao et al. reported that ergothioneine (a naturally occurring _L_-His derivative containing a sulfur atom on the imidazole ring) inhibited mushroom TYR activity (IC_50_ = 4.47 mM) in a competitive manner, whereas _L_-His exhibited no inhibitory effect [85]. 

Girelli et al. investigated the inhibitory activity of various glycyl-dipeptides (GD, GG, GH, GL, GK, GF, GP, GY) against mushroom TYR activity [86]. Most of the tested dipeptides, except for GP and GL, exhibited an inhibitory effect on TYR activity, with GD being the most active. Dipeptides GD, GK, and GH diminished the browning of fresh Golden Delicious apples and Irish White Skinned potatoes. Tseng et al. estimated the inhibitory capacity of 20 × 20 dipeptides against mushroom TYR [87]. Cys-containing dipeptides exhibited highly potent TYR inhibition, and N-terminal Cys-containing dipeptides, such as CE (IC_50_ = 2.0 μM), outperformed C-terminal Cys-containing dipeptides. Of the dipeptides (CA, YC, PD, and DY) tested in cells, PD reduced melanin content (16.5% reduction at 100 μM; 28.5% at 400 μM), whereas the Cys-containing CA and YC dipeptides exhibited weaker activities. 

Hsiao et al. used a pharmacophore modeling method to identify crucial complementary functional groups essential for mushroom TYR inhibition [88]. They identified active compounds A5 and B16, which resemble the chemical structures of the peptides WY and KFY, respectively, indicating that the C-terminal _L_-Tyr residue is important for TYR inhibition. Of the tripeptides tested, RCY and CRY exhibited high inhibitory activity against mushroom TYR. CRY containing an _L_-Cys residue at its N-terminus showed the more potent TYR inhibitory activity (IC_50_ = 6.16 μM) compared with kojic acid (IC_50_ = 84.4 μM) and arbutin (IC_50_ = 1008.7 μM). Luisi et al. compared the TYR inhibitory effects of a series of sulfurated amino acids and tripeptides [89]. In particular, _L_-Cys, _L_-Cystine, H-Glo(Cys-Gly-OH)-OH (the γ-oxa-glutamyl (Glo) analog of glutathione), and ergothioneine exhibited higher activity compared with glutathione (H-Glu(Cys-Gly-OH)-OH), whereas taurine exhibited a slightly weaker activity on a mass concentration basis.

Abu Ubeid et al. screened an internal library and identified active oligopeptides that inhibited mushroom TYR [90]. In particular, the oligopeptides, YRSRKYSSWY (IC_50_ = 40 μM) and RADSRADC (IC_50_ = 123 μM), were more active compared with hydroquinone (IC_50_ = 680 μM). Other peptides including KFEKKFEK (IC_50_ = 3.6 mM) and SFLLRN (IC_50_ = 8 mM) were less active. The peptides YRSRKYSSWY and RADSRADC also inhibited human TYR more effectively compared with hydroquinone. Treatment of human melanocytes with the peptides YRSRKYSSWY and RADSRADC at 100 μM for seven days reduced melanin content by 43% and 27%, respectively. This research group also performed a docking study using a library of short sequence oligopeptides against the crystal structure of mushroom TYR and identified a number of oligopeptides that exhibited favorable binding free energies and direct interaction with the catalytic pocket of the enzyme [91]. The mushroom TYR inhibitory activity of the identified peptides, RRWWRRYY (IC_50_ = 238 μM), RRRYWYYR (IC_50_ = 398 μM), and RRYWYWRR (IC_50_ = 282 μM), were more potent compared with hydroquinone (IC_50_ = 560 μM). The peptides showed a competitive mechanism of inhibition. The oligopeptides also inhibited human TYR activity and exhibited no cytotoxicity in melanocytes, keratinocytes, or fibroblasts up to 3 mM. However, it was not determined whether they exhibited antimelanogenic effects in melanocytes.

Park et al. reported that _D_-Tyr inhibited TYR activity by a competitive mechanism and reduced melanin content in human MNT-1 melanoma cells and primary human melanocytes stimulated by α-MSH or UV radiation [92]. Treatment with 10 mM _D_-Tyr reduced melanin synthesis in the epidermal basal layer of a 3D human skin model [92]. In a subsequent study, this research group demonstrated that the addition of a _D_-Tyr residue to the C-terminus of certain functional peptides could increase their TYR inhibitory activity in vitro and antimelanogenic activity in cells, while retaining the intrinsic properties of the unmodified peptides [93]. 

Arjinpathana et al. showed that oral administration of glutathione resulted in the lightening of human skin color [94]. Watanabe et al. reported that topical treatments of glutathione disulfide showed skin depigmenting effects [95]. Thus, both the reduced and oxidized form of glutathione can reduce melanin levels in the skin, probably by increasing pool of sulfhydryl compounds in melanocytes.

### 5.2. TYR Inhibitory Peptides Derived from Natural Protein Sequences

Various peptides derived from natural protein sequences inhibit TYR activity and display antimelanogenic effects in cells (Table 3). 

Morita et al. discovered several cyclic peptidic compounds from the roots of *Pseudostellaria heterophylla* that exhibited mushroom TYR inhibitory activity [98,99]. Of these compounds, cyclo[GGYLPPLS] (IC_50_ = 50 μM), cyclo[GTLPSPFL] (IC_50_ = 63 μM), and cyclo[PFSFGPLA] (IC_50_ = 75 μM) exhibited more potent inhibitory effects compared with the other compounds and arbutin (IC_50_ = 1.2 mM). These compounds represent rare examples of naturally occurring TYR inhibitory peptides.

Many natural proteins and peptides derived from milk, egg, wheat, rice, and vigna have been demonstrated to exhibit TYR inhibitory activity. Nakajima et al. examined the effects of whey proteins from bovine milk on melanogenesis in cultured human melanocytes [105]. Of the primary milk protein components, only β-lactoglobulin exhibited a significant depigmenting effect at a concentration of 1 mg/mL, whereas α-lactalbumin, serum albumin, and IgG showed no effect. β-Lactoglobulin decreased cellular TYR activity and reduced cell pigmentation induced by retinol and retinoic acid. Hernandez-Ledesma et al. reported that the β-lactoglobulin-derived peptide fragments, YFYPEL, WYSLAMAA, YVEEL, and MHIRL showed potent free radical scavenging activity against 2,2′-azobis (2-methylpropionamide) dihydrochloride [106]. Schurink et al. screened a large peptide library composed of octameric peptides from various industrial protein sources, including milk (β-casein, α-lactalbumin, and β-lactoglobulin), egg (ovalbumin), and wheat (gliadin, glycinin, and β-conglycinin), in order to identify peptides capable of inhibiting mushroom TYR activity [100]. As a result, they identified several TYR-inhibiting peptides, including MMSFVSLL and VSLLLVGI from α-lactalbumin and LILVLLAI from gliadin. They concluded that the presence of hydrophobic, aliphatic residues, such as Val, Ala, or Leu, is important for the TYR inhibition activity observed with these peptides.

Ochiai et al. prepared hydrolysates of rice bran protein by simultaneous treatment with chymotrypsin and trypsin, and identified several peptides that inhibited the monophenolase activity of mushroom TYR. These included LQPSHY (IC_50_ = 156 μM), HGGEGGRPY, and HPTSEVY in the order of decreasing activity. The peptide LQPSHY at 125–500 μM, but not the other two peptides, inhibited melanogenesis in mouse B16 melanoma cells without causing cytotoxicity [101]. Kubglomsong et al. compared the TYR inhibition activity of rice bran protein fractions, including albumin, globulin, glutelin, and prolamin, and found that the albumin fraction exhibited higher activity compared with the other protein fractions [102]. They also hydrolyzed rice bran albumin with papain and compared the TYR inhibition and copper chelation activities of the peptide fractions. Of the peptides from the rice bran albumin hydrolysate, SSEYYGGEGSSSEQGYYGEG showed the highest TYR inhibition activity (IC_50_ = 1.31 mg/mL), which was between that of citric acid (IC_50_ = 9.38 mg/mL) and ascorbic acid (IC_50_ = 0.03 mg/mL). This peptide also exhibited copper-chelating activity (IC_50_ = 0.62 mg/mL) which was stronger than that of ethylenediaminetetraacetic acid (EDTA) (IC_50_ = 1.06 mg/mL). 

Shen et al. reported TYR inhibitory activity of the ECGYF peptide, which consisted of a short sequence of the protein midasin found in Vigna [103]. The TYR inhibition activity of ECGYF (IC_50_ = 0.46 mM) was greater compared with that of arbutin and glutathione. The peptide ECGYF (0.5 – 1 mM) reduced melanin content in cultured A375 melanoma cells more effectively compared with arbutin or glutathione without exhibiting cytotoxic effects. This peptide also exhibited potent free radical scavenging activity against hydroxyl and superoxide radicals in vitro.

Some antimicrobial peptides were reported to have TYR inhibitory activity. Leucrocin I (NGVQPKY) is an antimicrobial peptide originating from crocodile white blood cell extracts [107]. Joompang et al. reported the mushroom TYR inhibitory activity of leucrocin I (IC_50_ >200 μM) and its modified peptides, NGVQPKC (IC_50_ = 132 μM) and CNGVQPK (IC_50_ = 113 μM), were relatively weaker compared with kojic acid (IC_50_ = 26 μM) [104]. Lineweaver–Burk plots indicated that leucrocin I (NGVQPKY) is a mixed type inhibitor, whereas NGVQPKC and CNGVQPK are competitive inhibitors. When B16F1 melanoma cells were treated with these peptides up to 350 μM, the greatest reduction of melanin content was observed with CNGVQPK, followed by NGVQPKC and leucrocin I (NGVQPKY). The melanin decreasing activity of CNGVQPK was similar to that of kojic acid. 

### 5.3. TYR Inhibitory Peptides Conjugated with Other Chemical Moieties 

Some amino acids and peptides have been hybridized with other antimelanogenic compounds, such as kojic acid, protocatechuic acid, α-resocylic acid, gentisic acid, gallic acid, caffeic acid, *para*-coumaric acid, and ascorbic acid to improve their activity, stability, or bioavailability (Table 4). 

In 2007, Noh et al. synthesized various hybrid compounds between kojic acid and tripeptides with a C-terminal carboxylic acid group or amide group [108]. The kojic acid-tripeptide amides showed enhanced stability at elevated temperature (50 °C) and, in an acidic solution (pH 4.8 and pH 5.8), although their inhibitory activity against mushroom TYR was similar to that of hybrid compounds between kojic acid and tripeptide acids. Of the hybrid compounds tested, kojic acid-FWY (IC_50_ = 1.28 μM), kojic acid-FHY (IC_50_ = 4.55 μM), kojic acid-FRY (IC_50_ = 5.92 μM), kojic acid-FWY-NH_2_ (IC_50_ = 2.2 μM), kojic acid-FHY-NH_2_ (IC_50_ = 2.36 μM), and kojic acid-FRY-NH_2_ (IC_50_ = 3.59 μM) exhibited higher inhibitory activity against mushroom TYR compared with kojic acid (IC_50_ = 94 μM). This research group also conjugated kojic acid with various amino acid amides and compared their TYR inhibitory activities [109]. Of these hybrid compounds, kojic acid-Phe-NH_2_ showed the highest inhibitory activity (IC_50_ = 14.7 μM), whereas kojic acid-Cys-NH_2_ exhibited the lowest. Kojic acid-Phe-NH_2_ was determined to be a non-competitive inhibitor by kinetic analysis and the inhibition mechanism was supported by docking simulation data. No data from cell experiments were presented. 

Singh et al. also synthesized five types of peptides conjugated with kojic acid, including kojic acid-PS, kojic acid-ECG, kojic acid-KECG, kojic acid-PKEK, and kojic acid-CDPGYIGSR [110]. Of these hybrid peptides, kojic acid-PS showed the most potent inhibitory effect against mushroom TYR activity (IC_50_ = 30 μM), followed by kojic acid-CDPGYIGSR (IC_50_ = 70 μM). Kojic acid-PS also attenuated melanin synthesis at 2–5 mM in cultured B16F10 mouse melanoma cells stimulated by α-MSH without exhibiting cell toxicity. 

In 2011, Noh et al. prepared hybrid compounds conjugating different aromatic amino acids, such as _L_-Phe, _L_-Trp and _L_-Tyr, to different hydroxyphenolic acids, including protocatechuic acid, α-resocylic acid, gentisic acid, and gallic acid [111]. Of these hybrid compounds, protocatechuic acid-amino acid amides showed potent TYR inhibitory activity. Protocatechuic acid-Phe-NH_2_, protocatechuic acid-Trp-NH_2_, and protocatechuic acid-Tyr-NH_2_ inhibited the diphenolase activity of mushroom TYR (IC_50_ = 0.56–0.66 mM). Of these compounds, protocatechuic acid-Phe-NH_2_ reduced melanin synthesis in B16 cells most effectively (56% inhibition at 100 μM). 

Yang et al. synthesized β-lactoglobulin fragment peptides conjugated with caffeic acid, including caffeic acid-MHIR, caffeic acid-HIRL, and caffeic acid-HIR [112]. The inhibitory activities of these peptide conjugates against mushroom TYR were higher compared with that of kojic acid. Caffeic acid-MHIR exhibited the highest TYR inhibition activity (IC_50_ = 47.9 μM), and it was determined to be a non-competitive inhibitor in a Lineweaver−Burk plot. All peptides including caffeic acid-MHIR did not show cytotoxicity toward B16-F1 melanoma cells at 100 μM, although changes in melanin content were not presented in this study. 

Park et al. compared various caffeic acid- and *para*-coumaric acid-conjugated peptides for their TYR inhibitory effects in vitro and their antimelanogenic effects in SK-MEL-2 human melanoma cells stimulated by α-MSH [113]. Of the conjugated peptides tested, *para*-coumaric acid-GGG-ARP inhibited TYR activity the most. It also reduced melanin content in cells and downregulated the expression of TYR, TYRP1, TYRP2, and MITF mRNA. Choi et al. synthesized ascorbic acid-KTTKS hybrid peptides. These compounds were significantly more stable compared with ascorbic acid and retained the antimelanogenic and collagen biosynthesis stimulating properties of ascorbic acid [114].

In our previous studies, *para*-coumaric acid was shown to be a potent inhibitor of TYR [115,116], and its antimelanogenic effects were verified in several in vitro and in vivo studies [117,118,119]. In addition, ascorbate coumarates, which are hybrid compounds between *para*-coumaric acid and ascorbic acid, were shown to inhibit melanin synthesis in epidermal melanocytes while increasing collagen synthesis in dermal fibroblasts [120]. Thus, both *para*-coumaric acid and ascorbic acid moieties are useful in making hybrid compounds with antimelanogenic activity. 

### 5.4. Peptides That Inhibit TYR Gene Expression 

Some peptides are known to downregulate TYR expression by acting as a MC1R antagonist or by other mechanisms (Table 5).

Various MSH fragment analogues have been designed and tested for their antagonistic activity in non-mammalian models, such as fishes, frogs, and lizards [127,128]. A peptide analogue H-His-_D_-Arg-Ala-Trp-_D_-Phe-Lys-NH_2_, designed based on the primary sequences of a growth hormone-releasing peptide and α-MSH, demonstrated the α-MSH antagonistic efficacy, attenuating the response to α-MSH or [Nle^4^,_D_-Phe^7^]-α-MSH in the lizards [61,121]. Many MSH-derived peptide analogs showed agonistic or antagonistic or no activity depending on the species, and their effects on TYR gene expression melanin synthesis in mammalian cells are not well established.

Choi et al. reported on the antimelanogenic effects of a disulfanyl peptide, which is a homo dimer of dipeptides containing Cys and Met residues connected by an intramolecular disulfide bond [122]. The peptide had no effect on TYR catalytic activity in vitro but decreased cellular levels of TYR and MITF while inducing the prolonged activation of extracellular signal-regulated kinase (ERK). The peptide-induced downregulation of MITF was abrogated by ERK inhibition with PD98059, G-protein coupled receptor inhibition with pertussis toxin, and lysosome inhibition with chloroquine, but not by proteasome inhibition with MG132. This indicated that the peptide reduced melanin synthesis by receptor-mediated, ERK-dependent suppression of MITF, resulting in downregulation of TYR in cells.

Lee et al. reported that a hexapeptide, SFKLRY-NH_2_, exhibited antioxidant effects in human dermal fibroblasts and antimelanogenic effects in B16 cells [123]. Prior to this study, the peptide had been originally identified to increase intracellular calcium in MS-1 mouse endothelial cells by another research group using a positional scanning substrate combinatorial library (PS-SCL) [129]. The SFKLRY-NH_2_ peptide induced proliferation, migration, and tube formation in human umbilical vein endothelial cells (HUVECs), supporting its angiogenic activity [129].

The PS-SCL was previously used to identify optimized sequences of peptide ligands and protease substrates [130,131]. In the case of the hexapeptide combinatorial libraries, 20 amino acids were incorporated at each of six diversity positions, resulting in 20^6^ individual peptides. In the PS-SCL format, the same diversity was arranged into six sub-libraries × 20 peptide pools. Each of the six positional sub-libraries (OXXXXX, XOXXXX, XXOXXX, XXXOXX, XXXXOX, and XXXXXO) consisted of 20 types of peptide pools. Each peptide pool has a fixed amino acid residue at the O position and an equimolar mixture of all 20 amino acids at the X position. Thus, in principle, the activity differences between the 20 peptide pools in a single positional sub-library is related to the different amino acids at the O position.

We used PS-SCL to identify antimelanogenic peptides [124]. Initial experiments, in which antimelanogenic activity of the peptide pools was evaluated in B16-F10 cells stimulated by α-MSH, predicted the active hexapeptide sequences as (I/F)-(N/S)-H-(H/N)-L-G-NH_2_ [124]. Additional experiments confirmed the antimelanogenic activity of the individual hexapeptides, including INHHLG-NH_2_, ISHHLG-NH_2_, INHNLG-NH_2_, ISHNLG-NH_2_, FNHHLG-NH_2_, FNHNLG-NH_2_, and FSHNLG-NH_2_ [124]. Of these peptides, FSHHLG-NH_2_ was the most active and it was ten times more active than arbutin. FSHHLG-NH_2_ reduced melanin synthesis and TYR expression in B16 cells stimulated by α-MSH or forskolin, and it also exhibited antimelanogenic effects in human melanocytes stimulated by _L_-Tyr or α-MSH. 

**Figure 2:**
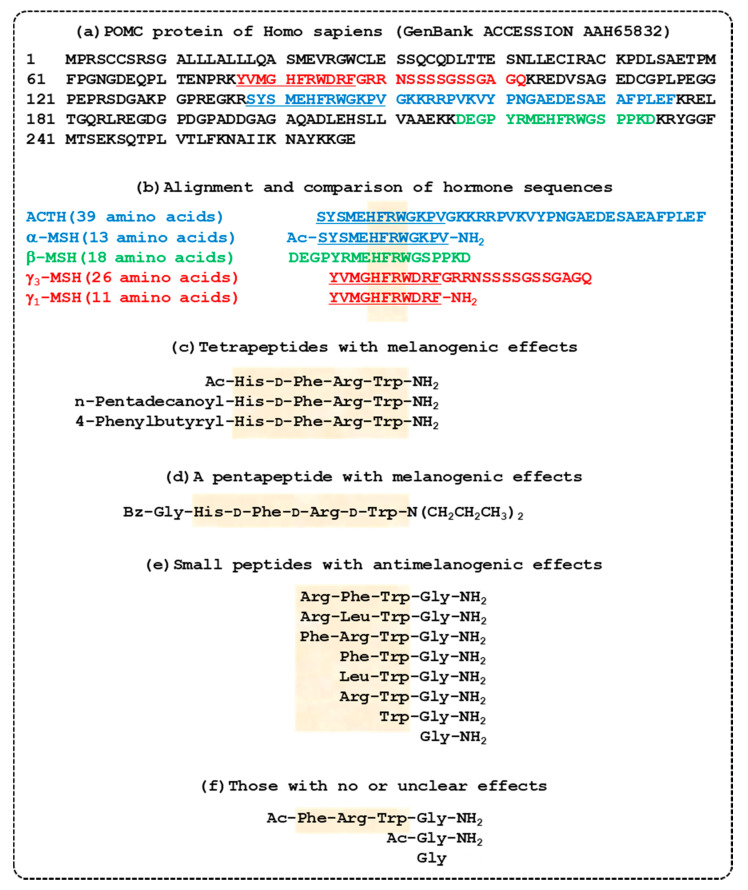
Sequences of proopiomelanocortin (POMC)-derived peptide hormones and synthetic peptides with melanogenic or antimelanogenic effects. (**a**) The entire amino acid sequence of the human POMC protein is shown. Sequences for different POMC-derived hormones are indicated with different colors: adrenocorticotrophic hormone (ACTH) in blue; α-melanocyte stimulating hormone (MSH) in underlined blue; β-MSH in green; γ_3_-MSH in red; and γ_1_-MSH in underlined red. (**b**) Amino acid sequences of ACTH, α-MSH, β-MSH, γ_3_-MSH, and γ_1_-MSH including posttranslational modifications are shown. A conserved sequence, His-Phe-Arg-Trp, is highlighted. (**c**) Tetrapeptides that stimulate melanin synthesis [62]. (**d**) A pentapeptide that stimulates melanin synthesis [63]. (**e**) Tetra-, tri-, di-, and mono-peptides that inhibit melanin synthesis [125]. (**f**) Molecules with no or unclear effects on melanin synthesis [61,125].

In a subsequent study aiming to identify antimelanogenic tetrapeptides using PS-SCL, the active sequences were predicted to be R-(F/L)-(C/W)-(G/R)-NH_2_ [125]. Of the individual tetrapeptides, RFWG-NH_2_, RLWG-NH_2_, and FRWG-NH_2_ showed high antimelanogenic activity. The tripeptides FWG-NH_2_, LWG-NH_2_, and RWG-NH_2_ were more active compared with RFW-NH_2_, RFG-NH_2_, RLG-NH_2_, or RLW-NH_2_, suggesting that the C-terminal WG-NH_2_ moiety is important for antimelanogenic activity. The dipeptide WG-NH_2_ and the monopeptide Gly-NH_2_ (glycinamide) also retained antimelanogenic activity, while Ac-Gly-NH_2_ and _L_-Gly were inactive. These antimelanogenic peptides have amino acid sequences similar to a part of α-MSH (Ac-SYSMEHFRWGKPV-NH_2_), thus it is thought that these peptides can target MC1R [41]. The antimelanogenic activity of Gly-NH_2_ is approximately 10 times and 22 times more potent than that of arbutin when compared based on molar concentration and mass concentration, respectively [125]. Sequences of POMC-derived peptide hormones and synthetic peptides with melanogenic or antimelanogenic effects are shown in Figure 2. 

Glycinamide hydrochloride (Gly-NH_2_⋅HCl) suppressed the activation of CREB and the mRNA expression of MITF and TYR in response to α-MSH, resulting in lower melanin synthesis [125]. In a subsequent clinical study, performed in a double-blinded format for eight weeks in 21 human subjects, a preparation containing 10% glycinamide hydrochloride showed a significant depigmentation effect without any noted adverse effects on the skin, compared with the control preparation without glycinamide hydrochloride [126]. This very small molecule has great potential to be used as a skin depigmentation agent. 

### 5.5. Peptides That Inhibit Melanosome Biogenesis or Induce Autophagy in Melanocytes

A few peptides are known to display antimelenogenic effects in melanocytes through modulation of melanosome biogenesis and autophagy (Table 6). 

Melanosome biogenesis can be a target for the tuning of skin pigmentation [10]. The interaction of heterotetrameric adaptor protein-1 (AP-1) and KIF13A, a microtubule motor protein, is an essential step for the sorting and trafficking of TYR and TYRP1 to melanosomes [134]. AP-1 recognizes and captures TYR and TYRP1 through specific motifs, and its β1-adaptin subunit interacts directly with KIF13A that transports the captured enzymes to the melanosomes. 

Using a peptide mapping strategy, Campagne et al. identified β1-adaptin-derived undecapeptides, EPLNNLQVAVK and QTVEISLPLST, which inhibited the binding of AP-1 to KIF13A in HeLa or MNT-1 cells [132]. Of the several pentapeptides derived from the active undecapeptides, pentapeptide QVAVK exhibited the most potent activity. It also inhibited the maturation of melanosomes. Undecapeptide EPLNNLQVAVK and pentapeptide QVAVK reduced melanin content in human MNT-1 cells and tripeptide QVA reduced melanin content in a three-dimensional reconstructed epidermis model. Thus, it is suggested that pigmentation can be controlled by the intervention of melanosome biogenesis and transport processes.

Autophagy is a lysosome-dependent mechanism that removes misfolded or damaged proteins and unnecessary organelles [135]. Kim et al. reported that a synthetic peptide derivative PTPD-12, which was originally developed as an activator of NAD-dependent deacetylase sirtuin-1, induced autophagy in human melanocytes, and even in keratinocytes that contained transferred melanosomes [133]. Autophagy induction in melanocytes by this peptide resulted in melanosome degradation and decreased melanin content without affecting the expression of MITF and melanogenesis pathway proteins. Topically applied PTPD-12 induced depigmentation in human skin explants. This study suggests that the modulation of autophagy may be a novel target for the regulation of skin pigmentation.

## 6. Discussion

This review introduced recent advances in the artificial regulation of skin pigmentation using amino acids, peptides, and their analogs. The most-studied molecular targets were the receptors on the surface of melanocytes which transmit intracellular signals, and the enzymes and proteins within melanocytes involved in melanin synthesis, and melanosome biogenesis and autophagy in melanocytes (Figure 1). 

_L_-Tyr was stimulatory, while _D_-Tyr and _L_-Cys were inhibitory to melanin synthesis in cells [76,84,92]. Many Cys-containing compounds, such as glutathione, inhibited TYR-catalyzed melanin synthesis by acting as a reactant for the thiol conjugation reaction with DOPAquinone as well as acting as an enzyme inhibitor [87,88,89]. A number of peptides from synthetic peptide libraries or natural sources exhibited inhibitory effects against TYR activity in vitro, and some showed antimelanogenic effects at the cell level [90,101,102,103,104]. Relatively high TYR inhibitory activities were achieved by hybrid compounds in which certain peptide sequences were conjugated with kojic acid [108,109,110], protocatechuic acid [111], caffeic acid [112,113], *para*-coumaric acid [113], or ascorbic acid [114]. 

Several peptides display more potent inhibitory effects against mushroom TYR activity and cellular melanin synthesis than other well-known inhibitors of melanogenesis, such as arbutin and kojic acid. CRY (IC_50_ = 6.16 μM) is more inhibitory than kojic acid (IC_50_ = 84.4 μM) and arbutin (IC_50_ = 1008.7 μM) [88]. Cyclo[GGYLPPLS] (IC_50_ = 50 μM), cyclo[GTLPSPFL] (IC_50_ = 63 μM), and cyclo[PFSFGPLA] (IC_50_ = 75 μM) are more inhibitory than arbutin (IC_50_ = 1.2 mM) [98,99]. Kojic acid-FWY (IC_50_ = 1.28 μM), kojic acid-FHY (IC_50_ = 4.55 μM), kojic acid-FRY (IC_50_ = 5.92 μM), kojic acid-FWY-NH_2_ (IC_50_ = 2.2 μM), kojic acid-FHY-NH_2_ (IC_50_ = 2.36 μM), and kojic acid-FRY-NH_2_ (IC_50_ = 3.59 μM) are more inhibitory than kojic acid (IC_50_ = 94 μM) [108]. Caffeic acid-MHIR (IC_50_ = 47.9 μM) is more inhibitory than kojic acid [112]. In cells, ECGYF reduces cellular melanin content more effectively than arbutin or glutathione [103]. CNGVQPK decreases cellular melanin content more effectively than kojic acid [104]. FSHHLG-NH_2,_ RFWG-NH_2_, RLWG-NH_2_, FRWG-NH_2,_ RFW-NH_2_, RFG-NH_2_, RLG-NH_2_, RLW-NH_2_, WG-NH_2_, and G-NH_2_ display very potent antimelanogenic activities in cells compared to arbutin [124,125]. Furthermore, there are lots of non-peptidic molecules, of which antimelanogenic effects were verified in cells [15,32,82,136,137,138] or in vivo [139,140,141,142]. Therefore, further studies are needed to examine the clinical utility of various peptidic molecules versus non-peptidic molecules for the treatment of skin pigmentary disorders.

The sequences of endogenous melanocortin hormones derived from the *POMC* gene product and numerous synthetic oligopeptides that showed melanogenic or antimelanogenic activity are shown in Figure 2. Even though γ_1_-MSH and γ_3_-MSH share a conserved sequence with other melanocortin peptides including ACTH, α-MSH, and β-MSH, they do not possess the pigmentary capabilities of their relatives [38,143]. Thus, it is inferred that the conserved sequence, His-Phe-Arg-Trp, renders a melanocortin peptide to be a potent agonist of MC1R, if the sequence is followed by a Gly residue (as in ACTH, α-MSH, and β-MSH). However, if the conserved sequence of a peptide is followed by an acidic Asp residue, the peptide may lose MC1R agonistic activity (as in γ_1_-MSH and γ_3_-MSH). 

It was shown that tetra- and pentapeptides containing a conserved sequence acted as potent agonists of MC1R [62,63]. These peptides may represent topically applicable alternatives to [Nle^4^-_D_-Phe^7^]-α-MSH, a stabilized analog of α-MSH [60]. In contrast, peptides with the sequence, Phe-Arg-Trp-Gly-NH_2_, or shorter sequences, did not show MC1R agonistic activities [125]. Instead, the peptide Phe-Arg-Trp-Gly-NH_2_, or shorter peptides that retained a Gly residue with an amide group at the C-terminus acted as an MC1R antagonist, preventing the melanogenic effects of α-MSH [125]. Of interest, while Gly-NH_2_ showed an antagonistic activity, neither Ac-Gly-NH_2_ nor Gly showed such activity [125]. In addition, while Phe-Arg-Trp-Gly-NH_2_ showed an antagonistic activity, Ac-Phe-Arg-Trp-Gly-NH_2_ rather potentiated the α-MSH-induced increase of TYR activity in murine melanoma cells, and did not show agonistic or antagonistic activity [61]. Therefore, it is suggested that small changes in the amino acid sequence of these peptides at conserved sequences or adjacent positions, and the presence of an acetyl group at the N-terminus or an amine group at the C-terminus result in significant differences in their effects on melanogenesis.

A variety of peptides and amino acid analogs were described to modulate melanin synthesis in cells, although their therapeutic utility remains to be further verified. In vivo and clinical results have been provided for MC1R targeting molecules, such as [Nle^4^-_D_-Phe^7^]-α-MSH [71,72], and glycinamide hydrochloride [126], and an inhibitor of melanin synthetic reaction, such as oxidized glutathione [95]. These studies suggest that certain amino acids, peptides, and their analogs may be a promising drug candidate for up- and downregulating skin pigmentation. Melanin increasing molecules can be used to alleviate photosensitive skin, to prevent photocarcinogenesis, and to treat vitiligo vulgaris [15,71,72]. Conversely, melanin decreasing molecules can be used to treat various types of hyperpigmentation for medical and aesthetic purposes [58,59,95,126]. 

Peptides exhibit a variety of advantageous properties, including low toxicity and fewer side effects compared with generic medicines, but they also display several disadvantages, such as low skin penetration and cell permeability and susceptibility to enzymatic degradation [144]. Large-sized or highly charged peptidic molecules have difficulty passing through the cell membrane and entering the melanosome to engage the target enzyme. Thus, the receptors on the surface of the plasma membranes of melanocytes, rather than the melanogenic enzymes inside melanosomes, represent more accessible targets for ‘cell-impermeable’ amino acids, peptides, and their analogs [145]. Otherwise, a strategy to target intracellular enzymes, proteins, or organelles using such peptides will require especially efficient delivery systems [146]. Although oral delivery is preferred, most peptide drugs are delivered intravenously or subcutaneously to avoid degradation in the gastrointestinal tract. Transdermal peptide delivery is also used, but it faces other problems including limited absorption. Therefore, research efforts are needed to overcome the inherent drawbacks of peptide drugs, including their poor pharmacokinetic properties. 

## 7. Conclusions

Probably because of the visually observable color change, the process of melanin synthesis catalyzed by several enzymes has provided a good model of enzyme research, thereby contributing to the advancement of biochemistry and biological science. The melanin synthesis process is also a good target for the discovery of peptide drugs because several amino acids and analogs participate as enzyme substrates and metabolites in the pathway, and several peptides are partly responsible for the fine-tuning of pigmentation in the skin. As we see in this review, many studies have been conducted to target the receptors on the surface of melanocytes, and the enzymes, proteins, and organelles within melanocytes that are involved in melanin synthesis, melanosome biogenesis, transport, and autophagy, using various types of amino acids, peptides, and their analogs. It is hoped that the research results so far will be the foundation for the development of excellent new peptide-based drugs that can be used for the treatment of skin pigmentary disorders. Additionally, it is hoped that this research experience in melanin biology will be utilized in targeting other metabolic processes that share similar regulatory mechanisms, contributing to the treatment of related diseases.

## Figures and Tables

**Table 1:** Amino acids, peptides, and their analogs that stimulate melanin synthesis.

Compounds	Key Points	Literature
[Nle^4^-_D_-Phe^7^]-α-MSH	This α-MSH analog was more resistant to enzymatic degradation and more potent in biological activity compared with α-MSH or [Nle^4^]-α-MSH.	[60]
Ac-Phe-Arg-Trp-Gly-NH_2_	This peptide enhanced the α-MSH-induced increase in TYR activity in S-91 murine melanoma cells.	[61]
Ac-His-_D_-Phe-Arg-Trp-NH_2_ n-Pentadecanoyl-His-_D_-Phe-Arg-Trp-NH_2_ 4-Phenylbutyryl-His-_D_-Phe-Arg-Trp-NH_2_	These tetrapeptides increased melanin synthesis and viability of human melanocytes under UV-irradiated conditions.	[62]
Bz-Gly-His-_D_-Phe-_D_-Arg-_D_-Trp-N(CH_2_CH_2_CH_3_)_2_	This pentapeptide induced protein expression of MITF, TYR, and TYRP1, and enhanced the activation of NRF2 after UVA-irradiation.	[63]
_L_-Tyr _L_-DOPA	_L_-Tyr and _L_-DOPA enhanced expression of TYR and stimulated melanin synthesis.	[64]
Vasoactive intestinal peptide (HSDAVFXDNYXRLRKQMAVKKYLNSXLN)	Vasoactive intestinal peptide increased melanin production by increasing TYR activity and gene expression in a PKA, CREB, and MITF–dependent mechanism.	[65]
Angiotensin II (DRVYIHPF)	Angiotensin II upregulated TYR activity and melanin content in melanocytes through an AT1-dependent mechanism.	[66]

**Table 2:** Amino acids, peptides, and their analogs that inhibit TYR activity and melanin synthesis.

Compounds	Key Points	Literature
_L_-Cys	_L_-Cys extended an initial delay in DOPAchrome formation by avocado and mushroom TYRs.	[84]
Ergothioneine	Ergothioneine inhibited mushroom TYR activity in a competitive manner, whereas _L_-His exhibited no inhibitory effect.	[85]
GD; GK; GH; GG; GF; GY	Glycyl-dipeptides such as GD, GK, and GH inhibited TYR activity, and reduced the browning of apples and potatoes.	[86]
CA; YC; PD; DY; CE; CS; CY; CW	Estimated TYR inhibitory activity of 20 × 20 dipeptides. N-terminal Cys-containing dipeptides were highly active.	[87]
CRY RCY	These antimelanogenic peptides were identified in a pharmacophore modeling method.	[88]
_L_-Cys _L_-Cystine H-Glu(Cys-Gly-OH)-OH H-Glo(Cys-Gly-OH)-OH Ergothioneine Taurine	_L_-Cys, _L_-cystine, H-Glo(Cys-Gly-OH)-OH, and ergothioneine inhibited TYR activity more strongly than glutathione (H-Glu(Cys-Gly-OH)-OH) and taurine.	[89]
YRSRKYSSWY RADSRADC KFEKKFEK SFLLRN	These oligopeptides were identified from an internal library and they inhibited TYR activity and reduced the melanin content of cells.	[90]
RRWWRRYY RRRYWYYR RRYWYWRR	These peptides were identified from a docking study against mushroom TYR and they were also inhibitory against the human TYR.	[91]
_D_-Tyr	_D_-Tyr inhibited TYR activity by a competitive mechanism and reduced melanin content in cells and a three-dimensional human skin model.	[92]
_D_-Tyr-_D_-Ala-Gly-Phe-Leu _D_-Ala-Gly-Phe-Leu-_D_-Tyr Gly-His-Lys-_D_-Tyr	The addition of _D_-Tyr to functional peptides endowed antimelanogenic activity without altering other bioactivities.	[93]
Glutathione	Oral administration of glutathione induced skin lightening of human volunteers.	[94]
Glutathione disulfide	Topical application of glutathione disulfide lowered melanin index in human skin.	[95]

**Table 3:** TYR inhibitory peptides derived from natural protein sequences.

Compounds	Key Points	Literature
Cyclo[GGYLPPLS] Cyclo[GTLPSPFL] Cyclo[PFSFGPLA]	These cyclic peptides from *Pseudostellaria heterophylla* inhibited TYR activity.	[98,99]
MMSFVSLL VSLLLVGI LILVLLAI	These antimelanogenic peptides were selected from octameric peptides with sequences of industrial proteins.	[100]
LQPSHY HGGEGGRPY HPTSEVY	LQPSHY derived from rice bran protein hydrolysates inhibited TYR activity and reduced melanin content in B16 cells.	[101]
SSEYYGGEGSSSEQGYYGEG	Of the peptides from the rice bran albumin hydrolysates, this peptide showed the highest TYR inhibition activity.	[102]
ECGYF	The peptide with a sequence of the protein midasin inhibited TYR activity and reduced melanin content in A375 melanoma cells.	[103]
NGVQPKY NGVQPKC CNGVQPK	These antimicrobial peptides inhibited TYR activity and reduced melanin content in B16F1 melanoma cells.	[104]

**Table 4:** TYR inhibitory peptides conjugated with other chemical moieties.

Compounds	Key Points	Literature
Kojic acid-FWY Kojic acid-FHY Kojic acid-FRY Kojic acid-FWY-NH_2_ Kojic acid-FHY-NH_2_ Kojic acid-FRY-NH_2_	These kojic acid-tripeptide amides showed enhanced stability and potent inhibition against TYR activity.	[108]
Kojic acid-F-NH_2_ Kojic acid-C-NH_2_	Of the kojic acid-amino acid amides, kojic acid-F-NH_2_ and kojic acid-C-NH_2_ showed the highest and lowest TYR inhibition, respectively.	[109]
Kojic acid-PS Kojic acid-CDPGYIGSR	These kojic acid-peptides inhibited TYR activity and reduced melanin synthesis in B16F10 cells.	[110]
Protocatechuic acid-F-NH_2_ Protocatechuic acid-W-NH_2_ Protocatechuic acid-Y-NH_2_	These hybrid compounds inhibited TYR activity and protocatechuic acid-F-NH_2_ reduced melanin synthesis in B16 cells most effectively.	[111]
Caffeic acid-MHIR	β-Lactoglobulin fragment peptides were conjugated with caffeic acid.	[112]
*para*-Coumaric acid-GGG-ARP	The compound inhibited TYR activity and decreased melanin content in cells.	[113]
Ascorbic acid-KTTKS	Ascorbic acid-KTTKS hybrid inhibited TYR activity and decreased melanin content in cells.	[114]

**Table 5:** Peptides that reduce TYR gene expression or its protein level in melanocytes.

Compounds	Key Points	Literature
H-His-_D_-Arg-Ala-Trp-_D_-Phe-Lys-NH_2_	This hybrid peptide analog derived from growth hormone-releasing peptide and α-MSH sequences demonstrated the antagonistic efficacy, attenuating the response to α-MSH or [Nle^4^,_D_-Phe^7^]-α-MSH in the lizards.	[61,121]
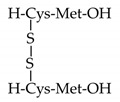	The tetrapeptide reduced melanin synthesis in cells by a receptor-mediated, ERK-dependent suppression of MITF and TYR expression.	[122]
SFKLRY-NH_2_	The peptide decreased TYR protein level in cells and showed antimelanogenic effects in B16 cells.	[123]
INHHLG-NH_2_ ISHHLG-NH_2_ INHNLG-NH_2_ ISHNLG-NH_2_ FNHHLG-NH_2_ FNHNLG-NH_2_ FSHNLG-NH_2_	These antimelanogenic hexapeptides were identified using PS-SCL. FNHHLG-NH_2_ reduced TYR expression and melanin synthesis in cells stimulated by α-MSH.	[124]
RFWG-NH_2_ RLWG-NH_2_ FRWG-NH_2_ FWG-NH_2_ LWG-NH_2_ RWG-NH_2_ WG-NH_2_ G-NH_2_	These low molecular antimelanogenic peptides with sequences overlapping with α-MSH inhibited melanin synthesis in cells stimulated by α-MSH. G-NH_2_ (glycinamide) attenuated phosphorylation of CREB and expression of MITF and TYR. Neither Ac-G-NH_2_ nor G showed antimelanogenic activity.	[125]
Gly-NH_2_•HCl	Glycinamide hydrochloride exhibited depigmenting effects without noted adverse effects in the human skin.	[126]

**Table 6:** Peptides and peptidic compounds that inhibit melanosome biogenesis or induce autophagy in melanocytes.

Compounds	Key Points	Literature
EPLNNLQVAVK QTVEISLPLST QVAVK QVA	Peptides derived from β1-adaptin inhibited the binding of AP-1 subunit to KIF13A, thereby inhibiting the maturation of melanosomes and melanin synthesis in cells.	[132]
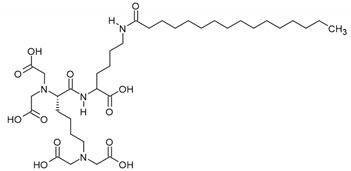	Pentasodium tetracarboxymethyl palmitoyl 21 dipeptide-12 induced autophagy in melanocytes and decreased pigmentation.	[133]

## Abbreviations

Ac-	Acetyl
AC	Adenylate cyclase
ACTH	Adrenocorticotrophic hormone
AP-1	Adaptor protein-1
ASP	Agouti signaling protein
AT1	Angiotensin II receptor type 1
Bz-	Benzoyl
cAMP	Cyclic adenosine monophosphate
CRE	cAMP response element
CREB	cAMP-responsive element-binding protein
DAG	Diacylglycerol
DCT	Dopachrome tautomerase
DHI	5,6-Dihydroxyindole
DHICA	5,6-Dihydroxyindole-2-carboxylic acid
DOPA	Dihydroxyphenylalanine
EDTA	Ethylenediaminetetraacetic acid
ERK	Extracellular signal-regulated kinase
GSK	Glycogen synthase kinase
MAPK	Mitogen-activated protein kinases
MC1R	Melanocortin 1 receptor
MCH	Melanin-concentrating hormone
MITF	Microphthalmia-associated transcription factor
MSH	Melanocyte-stimulating hormone
Nle	Norleucine
NO	Nitric oxide
NRF2	Nuclear factor erythroid 2-related factor 2
PKA	Protein kinase A
PKC	Protein kinase C
PLC	Phospholipase C
PMEL	Premelanosome protein
POMC	Proopiomelanocortin
PS-SCL	Positional scanning substrate combinatorial library
SCF	Stem cell factor
TYR	Tyrosinase
TYRP1	Tyrosinase-related protein 1
UV	Ultraviolet
VIP	Vasoactive intestinal peptide
